# Surgical Operations without Shock

**Published:** 1916-08-05

**Authors:** 


### SURGICAL OPERATIONS WITHOUT SHOCK.

### A Controverted Question of Technique.

The discovery of such anæsthetics as chloroform, ether, and the rest, marked a prominent departure in the practice of the art of surgery. It meant relief from suffering for countless individual patients, and, still more, it rendered easy the performance of many classes of operations which previously no surgeon had been able to contemplate. The next great step in surgical progress was the introduction of antiseptic surgery by Lister, and here again not only have innumerable lives been saved but the surgeon has been able to enter with impunity into regions of the body which had hitherto been closed to his enterprise. The general anæsthetic made possible the performance of the operation "in the absence of the patient," while by Listerism the risk of sepsis or blood-poisoning was reduced to a minimum. Thanks to these two agencies the surgical operation is deprived of a large part both of its terrors and of its risks, and even in the popular mind what was formerly regarded as a desperate adventure is now accepted as a reasonable and hopeful means of cure. There remains, however, and particularly in certain serious surgical undertakings, the risk of shock—the state of collapse or depression of the vital activities produced through the nervous system as a result of the manipulations or injuries which the surgeon is obliged to practise. This risk is not existent in all operations, but it is at times a real and even an urgent danger, and it is not wholly eliminated by the use of a general anæsthetic. This latter makes the patient unconscious of the violence inflicted directly or indirectly on his nervous system. But it does not abolish the other consequences of such violence. Hence the action of the heart and the state of the circulation may be interfered with by a surgical operation even though this is conducted under anæsthesia, just as similar interference may result from injury inflicted altogether apart from anæsthesia. Shock is therefore one of the bugbears of the surgeon, and many efforts have been made to avoid the chance of its occurrence.

Now as the state of shock is due to impulses travelling along the nerve trunks which lead from the area of the operation to the nerve centres, it follows that if these nerve trunks could be blocked —that is, could be rendered incapable of carrying impulses—shock would be prevented. At the same time the patient would feel no pain from the operation, for sensation, painful or otherwise, only occurs when nerve messages reach the brain, and this is impossible if the nerve trunks are rendered incapable of transmitting such messages. There are certain agents, such as novocaine, which, when injected under the skin, will produce this blocking or paralysing effect on nerve trunks. Hence when properly employed these drugs will render the part of the body to which they are applied insensitive, and a surgical operation can be performed entirely without the sensation of pain and also, it is claimed, without the production of shock. A state of *local* anæsthesia is produced, the other parts of the body retaining their normal qualities. The patient's consciousness remains unaffected because the nerve centres in his brain are not affected by the drug, which only influences the area where the local anæsthetic is applied. By such measures therefore it is possible to perform an operation while the patient watches the procedure and remains entirely free from pain. With increasing knowledge of the powers of these local anæsthetics more and more extensive operative procedures are being performed under their influence, and it is impossible to set a limit to what may be undertaken in this direction. The method avoids pain and avoids shock, and so far it is wholly to the good. But, clearly, it is not a pure advantage that while the surgeon is at work the patient should remain wholly awake and conscious of his surroundings. The mental strain upon him, especially in the case of some of the more serious and prolonged operations, would be very great, and the circumstances would be anything but helpful to the surgeon. The latter would no longer be operating "in the absence of the patient," which is one of the advantages of the use of a general anæsthetic. From all these considerations taken together has arisen the proposal to use both a local and a general anæsthetic, and so, as it were, to combine the advantages of both methods. The local anæsthetic prevents pain, and more particularly protects the patient from shock, while the general anæsthetic shuts off his consciousness from the whole of the proceedings by throwing him into a temporary sleep. Thus he neither feels pain nor suffers from shock, and he is unaware of the surgeon's proceedings. Such are the arguments advanced for the simultaneous use of a local and a general anæsthetic.

There are certain other advantages which are claimed for this combined method. When a general anæsthetic alone is employed it is necessary to administer it in relatively large amounts, so that the nerve centres may be completely dulled to the violent nerve messages which will be produced by the operation. Hence there are the risks which are attached to large doses, and a frequent after-effect is vomiting, which may be for the patient a source not only of discomfort but also of danger. When, however, by the use of a local anæsthetic the painful impulses excited by an operation are prevented from reaching the nerve centres it is only necessary to put these into a slight or moderate state of slumber. All that is necessary is to protect the patient from being aroused by the sights and sounds of the operating theatre, as from the pain of the operation he is already protected by the local anæsthetic. Hence a relatively small dose of the general anæsthetic is sufficient, and the disadvantages and dangers just described are therefore avoided. From this same consideration it becomes obvious that by the method of combined anæsthesia certain patients who would otherwise be beyond help may be brought within reach of surgical assistance. To give full doses of a general anæsthetic may be dangerous or even impossible to a patient who is the subject of, say, serious heart disease, and therefore when such a patient needs a severe surgical operation the surgeon may well shrink from its performance. But when the end can be attained by the use of the general anæsthetic in moderate amount the position is very different, and the most serious operations have been safely conducted, in the circumstances just mentioned, by the practice of combined anæsthesia. The actual achievement of the method means a great number of technical details, and to these surgeons are in an increasing degree now paying attention.

When the plea for combined anæsthesia is stated as above it certainly has a very attractive quality. But, as is the case with other therapeutic proposals, the final and conclusive test must be found not in theory but in practice. It is true that a certain number of surgeons have adopted the method and are enthusiastic in its praise. There are other authorities, however, who deliver a very different verdict, and some go so far as to condemn combined anæsthesia as both wrong in principle and bad in practice. Again, it is urged by some of the older school that the risks of shock are exaggerated and that these are fully met by such general anæsthetics as chloroform and ether, provided only that the administration is efficiently and skilfully conducted. These teachers contend that the light general anæsthesia advocated in the combined method is not sufficient for this end, and that mere blocking cannot be relied on to protect the brain from the effects of the nerve impulses set in motion by the operative procedures of the surgeon. They find, too, that a powerful element in the prevention of shock is related to the manner of the surgical manipulations, and that greater stress ought to be placed on delicacy of handling and a minimum of displacement of the tissues and organs exposed in the course of an operation. These latter points are advanced, also, by advocates of combined anæsthesia, so that apart from the main issue the controversy is not without its value. On this main issue the question for the present must remain an open one. No one ought to condemn in advance new proposals, but experience has many times shown that in medical and surgical practice the careful clinician guided by empirical observation is a safer guide than others who base their proposals largely on laboratory trials, and who, at least on paper, are able to make an admirable case for their claims. Hence the combined method of anæsthesia has its way to make against a large body of accumulated knowledge, and it cannot be said that so far it has gained anything like widespread professional confidence. The whole field of anæsthetics, curiously enough, has long been the site of much controversy, though it might have been anticipated that here in a very special degree unity of opinion and of practice would have existed. Only the other day an American anæsthetist argued that chloroform involves so many dangers that it ought to be absolutely and finally discarded, and according to the same writer spinal anæsthesia is so unsafe that its employment ought to be condemned. There is no royal road to the solution of these controversies. In part, doubtless, they are due to prejudices—by no means unwholesome prejudices—arising from personal experience and from a natural inclination to favour the tools which the individual anæsthetist has learned to handle. The inexperienced may bungle even with the best of methods, whereas the man of practice succeeds even although his proceedings are open to theoretical comment. What is to be desired is not the triumph of a particular doctrine or a particular school, nor is it indeed essential that uniformity of practice shall be established. The safety and welfare of the patient are the commanding consideration, and with this in view anæsthetists are perhaps the last body of practitioners who should be encouraged to run readily after some new thing. In a very direct sense the anæsthetist has human life in his keeping, and his responsibility is a very acute one. Those who have new practices to suggest in this field must be content to encounter a good deal of healthy conservatism, and some of them have to learn that change is not necessarily progress and that an intolerant disdain for established customs is not a tactful introduction with which to herald new proposals.

